# Design of Olmesartan Medoxomil-Loaded Nanosponges for Hypertension and Lung Cancer Treatments

**DOI:** 10.3390/polym13142272

**Published:** 2021-07-11

**Authors:** Bjad K. Almutairy, Abdullah Alshetaili, Amer S. Alali, Mohammed Muqtader Ahmed, Md. Khalid Anwer, M. Ali Aboudzadeh

**Affiliations:** 1 Department of Pharmaceutics, College of Pharmacy, Prince Sattam Bin Abdulaziz University, Alkharj 11942, Saudi Arabia; b.almutairy@psau.edu.sa (B.K.A.); a.alshetaili@psau.edu.sa (A.A.); a.alali@psau.edu.sa (A.S.A.); mo.ahmed@psau.edu.sa (M.M.A.); 2 Institut des Sciences Analytiques et de Physico-Chimie pour l’Environnement et les Matériaux, University Pau & Pays Adour, 64000 Pau, France

**Keywords:** ethylcellulose, encapsulation, lung cancer, nanosponge, oral bioavailability, systolic blood pressure

## Abstract

Olmesartan medoxomil (OLM) is one of the prominent antihypertensive drug that suffers from low aqueous solubility and dissolution rate leading to its low bioavailability. To improve the oral bioavailability of OLM, a delivery system based on ethylcellulose (EC, a biobased polymer) nanosponges (NSs) was developed and evaluated for cytotoxicity against the A549 lung cell lines and antihypertensive potential in a rat model. Four OLM-loaded NSs (ONS1-ONS4) were prepared and fully evaluated in terms of physicochemical properties. Among these formulations, ONS4 was regarded as the optimized formulation with particle size (487 nm), PDI (0.386), zeta potential (ζP = −18.1 mV), entrapment efficiency (EE = 91.2%) and drug loading (DL = 0.88%). In addition, a nanosized porous morphology was detected for this optimized system with NS surface area of about 63.512 m^2^/g, pore volume and pore radius Dv(r) of 0.149 cc/g and 15.274 Å, respectively, measured by nitrogen adsorption/desorption analysis. The observed morphology plus sustained release rate of OLM caused that the optimized formulation showed higher cytotoxicity against A549 lung cell lines in comparison to the pure OLM. Finally, this system (ONS4) reduced the systolic blood pressure (SBP) significantly (*p* < 0.01) as compared to control and pure OLM drug in spontaneously hypertensive rats. Overall, this study provides a scientific basis for future studies on the encapsulation efficiency of NSs as promising drug carriers for overcoming pharmacokinetic limitations.

## 1. Introduction

Globally, lung cancer is one of the leading causes of the cancer-related mortality and palliative care could bring about up the survival rate to 5 years for 15% of patients suffering from it [1]. Female breast cancer, colorectal, stomach and lung cancer make the 40% of the total cancer cases reported. Life-style modification shows an increase in the number of female smokers and smoking habits are relatively correlated for lung cancer due to the presence of mutagens in the inhaled smoke [2]. The prevalence of lung cancer is classically categorized into small cell lung cancer (SCLC) and non-small cell lung cancer (NSCLC) which is typically based on the histological features [3]. Among lung cancer patients, 85% exhibit non-small cell lung cancer (NSCLC) while the rest (15%) of the patients have small cell lung cancer (SCLC). Due to the fast metastasis and quick metabolism of SCLC, if left untreated, the mean survival rate would be about 120 days [4]. On the other hand, NSCLC is any type of epithelial lung cancer other than SCLC, which can be further, divided into lung adenocarcinoma (LUAD), broncho-alveolar, lung squamous cell carcinoma (LUSC), large cell carcinoma and bronchial carcinoid tumor [5]. Generally, cancer can be treated by surgery, radiation therapy, chemotherapy, immunotherapy, targeted therapy, hormone therapy, stem cell transplant, precision medicine and biomarker testing as suggested by the National Institutes of Health (NIH). Due to numerous limitations associated with these conventional methods, several researchers have exploited nanotechnology-based approaches for the efficient diagnosis and delivery of therapeutic agents [6]. In this context, nanomedicine has been applied successfully in developing novel nanocarriers, such as gold-nanoparticles, silver-nanoparticles, polymeric nanoparticles, nanoemulsion, self-emulsifying nanoemulsion and nanosponges [7].

Nanosponges (NSs) are porous nanocarriers with a particle size ≤1000 nm which is favorable for increasing the solubility, dissolution rate and sustained drug release action for temporal and targeted purposes [8]. The porous nature of the NSs could accommodate more drugs and diffusion of the solvent will be easy [9]. NS carriers are implicated in numerous disease conditions and early trials considered this nanotechnology five-folds more effective in comparison to conventional drug delivery systems, especially in malignant cancer treatment [10,11,12]. For example, it was shown in a study that the therapeutic efficacy of the anticancer drug was increased by the fabrication of ethylcellulose-based NSs containing brigatinib [13]. In another report, Zhang et al. introduced NSs as an antagonistic actuator to a viral mutation that successfully helped to neutralize the virus and inhibit SARS-CoV-2 infectivity [14]. Recently, we developed apremilast-loaded NSs as an efficient nanocarrier for the effective treatment of psoriasis and psoriatic arthritis condition. We observed that the pharmacokinetic profile of these carriers was increased 1.64-fold in terms of bioavailability compared to the pure apremilast suspension [8]. Moreover, in another study, we demonstrated that butenafine-loaded NSs could enhance the therapeutic efficacy through channeling the drug deeper into the skin layers at the target site to completely eradicate fungal infections [15]. In addition, starch derivatives and especially, cyclodextrin-based NSs have recently emerged due to the excellent properties owing to their distinct structure [16,17].

Drugs such as olmesartan medoxomil (OLM), which have been used in this study, have some intrinsic biopharmaceutical drawbacks that could be overcome by nanomedicine technology. Fast metabolism and poor water solubility of this drug result in its poor bioavailability (~28%). Along this portentous dissociation of OLM causes enteropathy due to direct facing of intestinal villi to free olmesartan that stimulate the cell-mediated immune reaction on prolong usage of this medicine [18,19]. Due to numerous therapeutic applications of OLM over commercially available antihypertensive drugs, formulators are exploring novel nanocarriers to improve the intestinal permeability, oral bioavailability and therapeutic effectiveness of OLM through its encapsulation in nanocarriers with the aim of achieving a sustained drug release pattern. PLGA nanoparticles [19,20], self-emulsifying drug delivery systems (SEDDS) [21,22], nanosuspension [23], nanocrystal [24] and other conventional and novel drug delivery systems [25,26] have been developed in this regard. It is worth remarking here that OLM exhibits anti-proliferative and anti-metastatic effects on tumors too [27]. For example, a study conducted in a mouse model by Vassiliou et.al [28] reported that OLM could reduce levels of plasminogen activator inhibitor-1 (PAI-1), whose high levels could cause oral cancer. Besides, another study reports the effectiveness of OLM against the A549 cell lines indicating lung cancer [29].

OLM, chemically named as 2,3-dihydroxy-2-butenyl4-(1-hydroxy-1-methylethyl)-2-propyl-1-[p-(o-1Htetrazol-5-ylphenyl)benzyl]imidazole-5-carboxylate, cyclic-2,3-carbonate, is a potent orally active angiotensin II receptor blocker. It is a prodrug that is hydrolyzed to the active olmesartan throughout absorption from the gastrointestinal tract (GIT) [30]. Angiotensin II receptor blockers are major regulators of blood pressure that competitively inhibit the binding of angiotensin II to its receptor. OLM is an angiotensin-II receptor antagonist (AIIRA) that acts selectively by binding angiotensin receptor 1 (AT1), thereby, preventing the protein angiotensin-II from binding, responsible for vasoconstriction. Physiologically, olmesartan reduces blood pressure (BP), cardiac activity, and aldosterone level and increases sodium excretion [31,32,33]. Hypertension is a high pressure exerted by circulating blood against the walls of the body’s arteries. If the measured pressure is ≥140 mmHg in the heart contract (systolic) and ≥90 mmHg for diastolic pressure at the heart rest, is considered as hypertension (HPT) also called as high blood pressure (HBP). HBP often referred as the silent killer with the prevalence of 972 million people globally accounted for about 26% of the world’s population which is expected to grow up to 29% by 2025. The global mortality of this disease is about 7.6 million deaths per year (13.5% of the total). The global antihypertensive drugs accounted for $26.3 billion in 2018 to $27.8 billion by 2023 with a compound annual growth rate (CAGR) of 1.1% for the period of 2018–2023 [34].

The primary aim of this work is to promote the oral bioavailability of OLM through its encapsulation in nanosponge carriers and secondly to investigate the ability of OLM-loaded ethylcellulose-based nanosponges to improve the antihypertensive and cytotoxicity activity against A549 lung cancer cell lines. The specific A549 cell lines were selected because OLM represents established in vitro experimental model to study the cytotoxicity potential of OLM [29]. Therefore, four different OLM-loaded nanosponges (ONS1-ONS4) were developed by varying the content of ethylcellulose polymer. Analyzing the formulations in terms of physicochemical properties and the encapsulation efficiency allowed us to select the optimized nanosponge carrier which was investigated further for antihypertensive activity and cytotoxicity against A549 lung cancer cell lines. The findings in this study may be further sculpted into new cancer and hypertension treatment strategies if this newly synthesized formulation induces desirable cytotoxic activity.

## 2. Materials and Methods

### 2.1. Materials

Olmesartan medoxomil (OLM) was purchased from “Mesochem Technology Co. Ltd., Beijing, China”. Ethylcellulose (EC), polyvinyl alcohol (PVA) and dichloromethane (DCM) were purchased from Sigma-Aldrich, St. Louis, MO, USA. All the other chemicals were of analytical grade.

### 2.2. Preparation of OLM-Loaded Nanosponges

**Figure 1:**
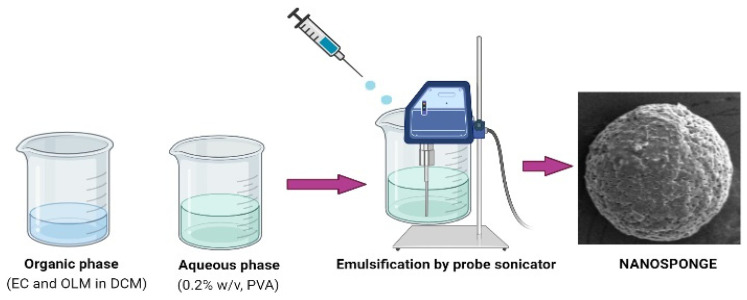
Schematic representation of nanosponge preparation.

OLM-loaded NSs were prepared by emulsion-solvent evaporation method [8]. Four NSs developed by dissolving EC (50–200 mg) and OLM (40 mg) in 5 mL of DCM and ultra-sonicated (Ultrasonic-Water Bath; Daihan Scientific, Model: WUG-D06H, Gangwon, Korea) for 3 min, after which this organic phase was emulsified by adding dropwise into 100 mL of aqueous phase (PVA, 0.2%, *w*/*v*) solution using probe-sonication (SONIC DISMEMBRATOR Model-FB120, Fisher scientific, Waltham, MA, USA) attached with probe (435 A) working at 60% power for 3 min (Figure 1). Thereafter, the organic solvent was evaporated by keeping the dispersion under stirring (Isotemp®, Fisher scientific, Model-DLM 1886×3, Waltham, MA, USA) over night at 700 rpm under atmospheric condition. Once the solvent was completely evaporated, the dispersion was centrifuged at 12,000 rpm, 25 °C for 15 min (Hermle Labortechnik GMBH, Model-Z216MK, Wehingen, Germany). The pellet of OLM-loaded NSs were then washed several times with milli-Q water to avoid adsorption of PVA on the surface of NSs. Stabilizer-free NSs were lyophilized (Millrock Technology, Kingston, NY, USA) and preserved for particle analysis and evaluations. The composition and precursors of the four NSs composition are tabulated in Table 1.

### 2.3. Measurement of Particle Size, Polydispersity Index (PDI) and Ζeta-Potential (ζP)

The size, PDI and ζP of OLM-loaded NSs were evaluated by dynamic light scattering (DLS) technique using Malvern-Nano ZS-Zetasizer, Malvern, UK. This instrument determines the particle size from intensity–time fluctuations of a laser beam (633 nm) scattered from a sample at an angle of 173°. To avoid multiple scattering, the samples under investigation were dispersed into Milli-Q water in (1:200) dilution concentration and ultra-sonicated (Ultrasonic-Water Bath; DaihanbScientific, Model: WUG-D06H, Gangwon, Korea) for three min in order to break the agglomerates and separate adherents. The sample is then filled in the disposable cuvettes (Folded capillary zeta cell—DTS1070) and kept in the machine with calibrated temperature of 25 °C [35]. Three measurements were performed with 100 cycles in each measurement for both size and PDI together followed by ζP.

### 2.4. Measurement of Percent Entrapment Efficiency (%EE) and Drug Loading (%DL)

An indirect method was followed in order to measure the %EE and %DL. The aqueous dispersion of NSs were centrifuged (Hermle Labortechnik GMBH, Model-Z216MK, Wehingen, Germany) in order to separate nanosponge particles, and supernatant was analyzed for free OLM drug by UV-spectroscopy (Jasco-UV-visible spectrophotometer, Model: V-630, Tokyo, Japan) at wavelength 255 nm. Each sample was analyzed chemically for drug content in supernatant (n = 3). The %EE and %DL of the OLM-loaded NSs were calculated using Equations (1) and (2), respectively [36].

(1) $$\%\mathrm{EE}=\frac{\mathrm{Amount}\;\mathrm{of}\;\mathrm{drug}\;\mathrm{added}-\mathrm{amount}\;\mathrm{of}\;\mathrm{drug}\;\mathrm{in}\;\mathrm{supernatant}\;}{\mathrm{Amount}\;\mathrm{of}\;\mathrm{drug}\;\mathrm{added}}\;\times \;100$$

(2) $$\%\mathrm{DL}=\frac{\mathrm{Amount}\;\mathrm{of}\;\mathrm{drug}\;\mathrm{in}\;\mathrm{nanosponge}\;}{\mathrm{Total}\;\mathrm{amount}\;\mathrm{of}\;\mathrm{polymer}\;\mathrm{added}}\;\times \;100$$

### 2.5. Fourier Transform Infra-Red (FTIR) Spectroscopy

The FTIR spectra of pure OLM and OLM-loaded NSs (ONS1-ONS4) were recorded using FTIR spectrometer (Jasco FTIR Spectrophotometer, Tokyo, Japan). Each samples were diluted with crystalline potassium bromide (sample:KBr, 1:10 *wt*/*wt*), and pressed into transparent film. The film was kept on sample holder and spectra was recorded using spectra manager software [37].

### 2.6. Differential Scanning Calorimetry (DSC) Studies

The DSC thermal spectra of pure OLM and their developed nanosponges (ONS1-ONS4) were recorded using a DSC instrument (N-650, Scinco, Seoul, Korea). Each sample (approx. 5 mg) was pressed in aluminum pan covered with a lid. The pressed pan was placed in DSC sample holder and heated in the temperature range of 25 to 200 °C at a rate of 20 °C/min with continuous purge of nitrogen gas during the analysis [37].

### 2.7. Powder X-ray Diffraction (PXRD) Studies

PXRD of pure OLM and their developed nanosponges (ONS1-ONS4) were recorded using a X-ray Diffractometer (Ultima-IV, Rigaku, Japan) in the range of 0–90° (2θ) at a scan rate of 4°/min. The PXRD spectra of each sample were taken at voltage and current 30 kV and 25 mA, respectively [37].

### 2.8. In Vitro Release and Kinetic Studies

In vitro release study was performed as reported based on a previous study [8]. Briefly, pure OLM (50 mg) and the optimized NS drug carrier (ONS4) were suspended in 5 mL of phosphate buffer (pH-6.8) and enclosed in diffusion semipermeable cellophane membrane (Hi-media Mol. 12,000 Dalton). The dialysis bag is then dipped into 50 mL of buffer medium, maintained at 37 °C under stirring at 50 rpm on thermostatically controlled magnetic stirrer (Isotemp®, Fisher scientific, Model-DLM 1886×3, Waltham, MA, USA). The samples were withdrawn (1 mL) and replaced with equal amount with the freshly prepared buffer at predetermined time intervals. The aliquots were analyzed spectrophotometrically at 255 nm (Jasco-UV-visible spectrophotometer, Model: V-630, made in Japan) [20]. The concentration was determined, and cumulative (%) release curve was plotted against time intervals (0, 0.5, 1, 2, 3, 4, 5, 6, 12 and 24 h). The release data were fitted to various release kinetic models, viz., zero order, first order, Higuchi and Krosmayer–Peppas kinetic models by imposing relation between % drug release vs. time, % log cumulative drug release vs. time, % log cumulative drug release vs. square root time and % log cumulative drug release vs. log time, respectively [36].

### 2.9. Scanning Electron Microscopy (SEM) Studies

Based on the physicochemical parameter and in vitro release studies, the NS (ONS4) was optimized and further examined for the surface morphology and nanocarrier diameter using SEM imaging (Joel JSM-SEM, model: JSM6330 LV, Tokyo, Japan). The lyophilized NSs (ONS4) was cautiously mounted on the SEM stubs and coated with Au (gold). Scanning was carried out at different magnifications and zones were captured and further processed to elucidate the surface property [37].

### 2.10. Nitrogen Adsorption/Desorption Characterization of OLM-Loaded NSs

The surface area, pore volume and pore radius of the optimized nanosponge drug carrier (ONS4) were analyzed by nitrogen sorption isotherm using Brunauer Emmett Teller (BET) method [38] (Quantachrome Instruments-version 5.0, Anton Paar, FL, USA), whereby the under investigation powdered sample was first put in the glass-bulb sample holder followed by heating overnight at 50 °C under negative pressure of 0.1 MPa in order to remove the moisture. Surface area was calculated by BET analysis from the nitrogen sorption data over a relative pressure (*P/PO*) of 1.003–5.047 range. However, the pore volume and pore radius were also obtained from the Barrett–Joiner–Halenda (BJH) summary data.

### 2.11. Cell Viability MTT Test

Lung cell lines A549 cells were seeded in 96-well plates (Thermo Scientific™ PCR Plate, 96-well, Waltham, MA USA) at a density of 5000 cell/well and were treated at different concentrations (6.25, 12.5, 25, and 50 µg/mL) of OLM-pure, ONS4 as well as the blank-NSs for 24 h. The cells added with the respective samples were treated with 10.0 µL of a 5.0 mg/mL MTT solution, incubated at 37 °C in presence of CO_2_. Precipitate of pre-washed PBS (phosphate buffer saline) was dissolved in 150 µL DMSO for 20 min and analyzed at 563 nm [39]. Cell viability (%) of the blank NS and the pure OLM and ONS4 carrier were compared for each concentration followed by processing the data for calculation of IC50.

### 2.12. In Vivo Antihypertensive Studies

The antihypertensive efficacy of the developed optimized nanosponge system (ONS4) was evaluated on spontaneous hypertensive rats (SHRs). The study protocol was reviewed and approved by “Animal Ethics Committee (Approval number: BERC 005-05-19), College of Pharmacy, Prince Satam Bin Abdulaziz University, Alkharj, Saudi Arabia”. The rats were divided into three groups (n = 6 per group) and they were kept for 7 days in controlled conditions, temperature (22 °C), RH (55 ± 5%), 12 h light/dark cycle and fed with standard diet with water ad libitum. Group I (control) received 0.5% *w*/*v*, sodium carboxy methylcellulose, group II received pure OLM drug suspension (1 mg/kg) and group III received the optimized nanosponge drug carrier (ONS4) (equivalent to 1 mg/kg of OLM). The rats were kept in a holder, pre-warmed and systolic blood pressure (SBP) of animals were measured using non-invasive tail cuff device (Kent Scientific Corp, Torrington, CT, USA) at 0 h, before the treatment and then at 0.5, 1, 2, 4, 6, 8, 10 and 12 h after treatment with drug and formulation [40].

### 2.13. Statistical Analysis

Results of physicochemical characterizations were expressed as the mean ± standard error of the mean (SEM). For in vivo antihypertensive studies, statistical variations of different treatment groups were analyzed according to one-way analysis of variance (ANOVA) followed by post-hoc Tukey’s test. *p* < 0.05 was considered statistically significant. Statistical analysis was performed using the GraphPad Prism program (version 4) (GraphPad Software, San Diego, CA, USA).

## 3. Results and Discussions

### 3.1. Measurement of Size, PDI and ζP

The particle size (PS), PDI and ζP of the developed OLM-loaded NSs (ONS1-ONS4) are presented in Table 2. The particle size of ONS1-ONS4 was measured in the range of 381–487 nm. The smaller (381 nm) and bigger size (487 nm) of OLM-loaded NS were measured for ONS1 and ONS4, respectively. It was observed that the size of NS was increased with the increase in amount of EC polymer. For encapsulating drugs, a small particle undoubtedly would be an advantage as it would carry only a small drug quantity, which will help in minimizing the onset of abrupt toxic response [41]. The PDI values of the NSs were measured in the range of 0.312–0.446, which is considered to be acceptable in drug delivery applications and indicates a homogenous population of particles. The aqueous dispersion was used to determine zeta potential in order to get stability of prepared NSs. The ζP of the OLM-loaded NSs were found in the range of −15.7 to −19.0 mV. It is believed that the values of ζP ≥ ±30 mV indicate formation of a stable dispersion, however it has to be remarked that the usual Smoluchowski method to calculate ζP is only valid for hard spheres. In this case, due to the soft nature of NSs, ζP calculated by conventional analysis does not reflect the state of agglomeration or stability. Our soft NS particles were stable despite ζP < ±30 mV [42].

### 3.2. Measurement of %EE and %DL

Generally, an ideal drug carrier should have high entrapment efficiency (EE). High EE (above 70%) can increase the efficacy of the drug delivery system and decrease the side effects of the drug [35,43,44]. The %EE and %DL of developed OLM-loaded NSs (ONS1-ONS4) were measured in the range of 86.6–91.2% and 0.88–1.89%, respectively (Table 2). Nanosponges can encapsulate drug up to three times to their weight due higher porosity [45]. The highest drug encapsulation was detected in ONS4, probably due to presence of large amount of EC polymer that prevents the leakage of drug from NSs, therefore, we selected this formulation as the optimized carrier system. Due to poor aqueous solubility, the OLM and ehylcellulose polymer were solubilized in DCM forming a viscous solution, which prevents the drug molecules from coming out to aqueous phase. That resulted in more drug encapsulation in polymeric system [46]. It is revealed that the more EC polymer content, the higher drug entrapment. As polymer content increased, the binding capacity or matrix forming ability of polymer with the drug also increased, due to this the maximum amounts of drug get entrapped in NSs producing more percentage of EE in higher drug to polymer ratio than lower ratio [47]. In this context, Maji et al. found that the drug %EE of EC microparticles surronding metformin HCl prepared by solvent-evaporation technique increased with increasing polymer concentration, when the surfactant content and the stirring speed were constant, increasing polymer content induced better coating onto the drug particles [48].

### 3.3. FTIR Studies

The interactions between drug and excipients were analyzed by comparing the FTIR spectra of the pure OLM and the obtained OLM-loaded nanosponges (ONS1-ONS4) (Figure S1). The FTIR spectra of pure OLM showed its sharp characteristic peaks assigned at 3289 cm^−1^ for O–H stretching vibrations, 2954 cm^−1^ for C–H stretching of aromatic rings, 1764 cm^−1^ for C=O stretching of the carboxylic group and 1173 cm^−1^ for C–N stretching vibrations, all these peaks were identical to the OLM confirming its purity [20]. Compared to the pure OLM, the characteristic peaks of OLM were present, weakened or shifted in OLM-loaded nanosponges (ONS1-ONS4). The FTIR interpretation of pure OLM and nanosponges (ONS1-ONS4) confirmed no significant interaction of OLM with excipient and therefore suggested successful entrapment and dispersion of OLM inside EC polymer [20]. From this spectral analysis, the OH and carbonyl groups were affected by possible host/guest hydrogen bond formation. In fact, when a carbonyl group and/or hydroxyl groups connect to a hydroxylic compound by hydrogen bonds, the stretching band shifts or weakens in intensity. This appears clearly in the spectrums of ONS1-ONS4 where there are a clear shift and fall in the intensity of the characteristic OH and C=O stretchings of the hydrate, respectively. Moreover, the characteristic peaks of OLM in the wavelenght range 1000 to 2000 cm^−1^ were weakened due to intermolecular interaction between C=O and C–N groups, resulting from higher electronegativity of oxygen and nitrogen. These findings stated that in different spectra of nanosponges no new peaks appeared which indicate that no chemical bonds were created in the developed formulations.

### 3.4. DSC Studies

**Figure 2:**
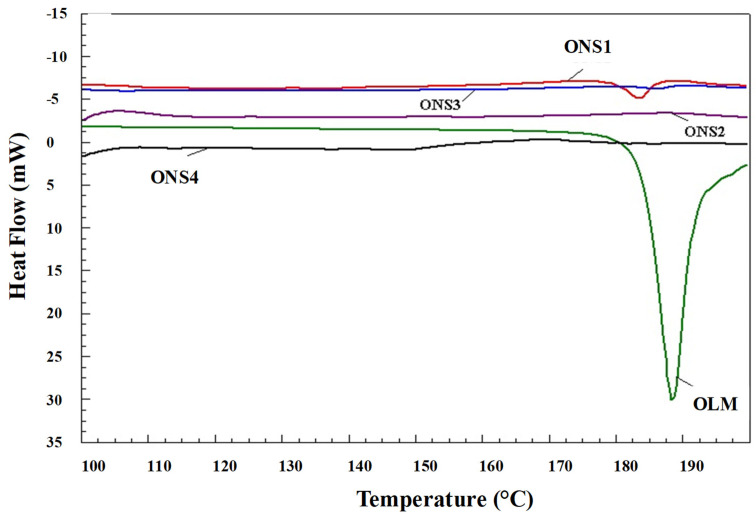
Comparative DSC spectra of OLM-loaded nanosponges.

DSC analysis confirmed the structural changes observed by FTIR measurements. DSC thermal behavior of the pure OLM and the developed nanosponges drug carriers (ONS1-ONS4) were presented in Figure 2. A sharp endothermic peak at 188 °C was observed in the free OLM drug, which was corresponded to the melting temperature of the drug [20]. A reduced intensity in endothermic peak could be seen in ONS1 formulation compared to the pure OLM, probably due to the less amount of EC used in formulating ONS1 nanosponges. However, in the systems ONS2, ONS3 and ONS4, complete disappearance of endothermic peak corresponding to 188 °C was observed, which was probably due to conversion of crystalline into amorphous OLM or molecularly dispersed into polymer matrix that could confirm complete encapsulation of drug inside EC polymer.

### 3.5. PXRD Studies

X-ray diffractograms of pure OLM and the developed OLM-loaded nanosponges (ONS1-ONS4) were presented in Figure S2. The XRD results strongly supported the data of DSC analysis. The XRD pattern of the pure OLM drug showed various intense peaks at 2θ values of 7.20°, 9.20°, 10.60°, 11.60°, 12.80°, 14.50°, 16.60°, 18.50°, 19.70°, 21.90°, 22.10°, 23.40°, 24.70°, 25.20°, 38.10°, 44.30° and 77.5°, confirming the crystalline nature of the drug [20]. However, the OLM-loaded nanosponges (ONS1-ONS4) were evaluated by weakening or disappearance of intense peaks when compared to the pure OLM drug. This clearly reveals conversion of crystals to amorphous form due to the dispersion of OLM drug in EC polymer. The polymeric encapsulation layers confine the drug film, which is then unable to crystallize at the solid–air interface. As a consequence, the coating layer introduces another solid–solid boundary. This process is called amorphous solid dispersion and is certainly the result of disrupting intermolecular interactions in the drug’s crystal lattice and forming drug–polymer interactions [49].

### 3.6. In Vitro Release and Kinetic Studies

**Figure 3:**
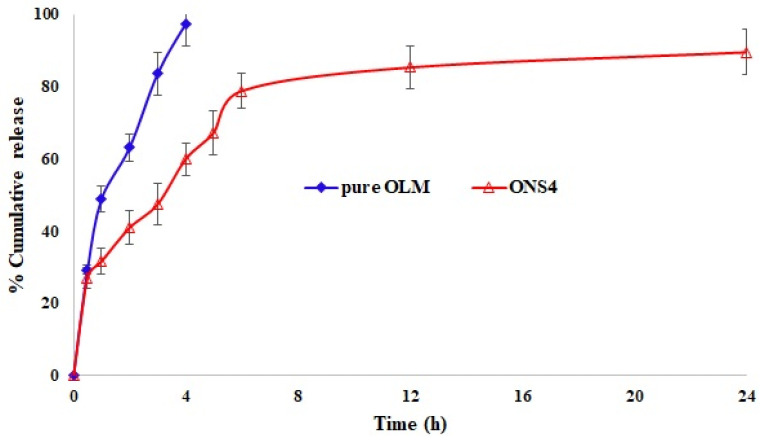
Comparative in vitro release profile of the pure OLM and the optimized nanosponges drug carrier (ONS4) at pH-6.8 and 37 °C after 24 h.

One of the important characteristics of drug delivery systems in biomedicine is to impart the sustained release of a drug [50]. Thus, the sustained drug release improves the accumulation of OLM at the tumor site while enhancing its anticancer performance. In vitro release profile of OLM and the optimized NS drug carrier (ONS4) are presented in Figure 3. The rate of OLM drug release depends on the content of EC polymer. ONS4 carrier showed the sustained release pattern, 89.5% of OLM was released in 24 h, probably due to viscous and swelling nature of EC polymer. In contrast, free OLM drug released 97.36% in the first 4 h. The decreased release rate of OLM-loaded NSs might be due to reduction of drug crystallinity (confirmed by PXRD studies), carrier solubilization effect, reduction of drug agglomeration, conversion of amorphous state and increased drug wettability. In addition, EC guarantees drug dissolution in entire gastrointestinal tract that maintains the constant release of drug for longer period of time and eliminates multiple dosing in a day; hence it improves the efficacy of drug [51]. Furthermore, EC is a non-toxic, hydrophobic, viscous and biocompatible polymer with good compressibility that make it suitable for developing sustained drug release formulations [51,52]. The in vitro release data were fitted to various kinetic models and highest correlation of coefficient (R^2^) was found for Higuchi model (R^2^ = 0.9908) (Figure S3), this reveals that the drug release mechanism is governed by diffusion process [53].

### 3.7. SEM Studies

**Figure 4:**
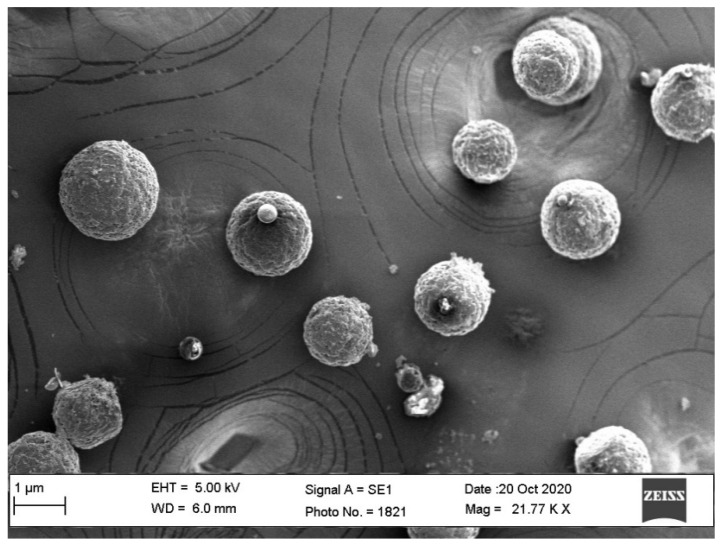
SEM images of the optimized nanosponge system (ONS4).

The SEM images of optimized NS (ONS4) are shown in Figure 4, which revealed a nanosized, spongy spherical NSs with a porous surface. Numerous tiny pores could be seen on the surface of nanosponge. The formation of pore was probably due to inward diffusion of DCM solvent [54]. The size of particles observed from SEM confirmed the findings observed by DLS method.

### 3.8. Nitrogen Adsorption/Desorption Characterization of OLM-Loaded NSs

**Figure 5:**
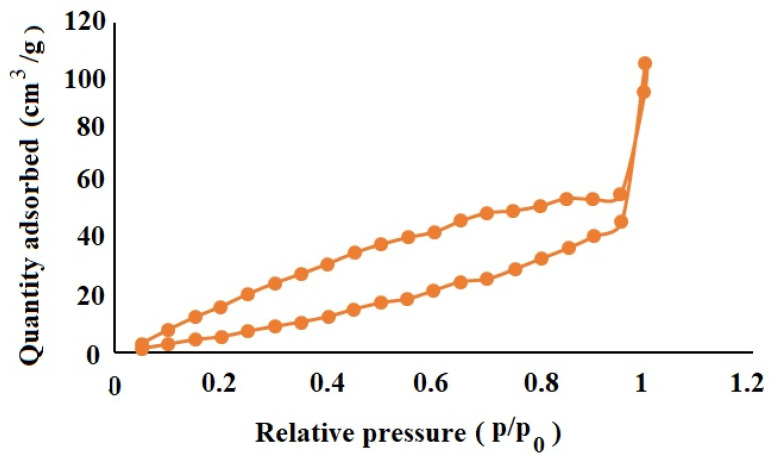
The Nitrogen adsorption–desorption isotherms for the optimized ONS4 system.

Nitrogen adsorption/desorption analysis of the optimized NS fomulation (ONS4) revealed a surface area of 63.512 m^2^/g, with pore volume and pore radius Dv(r) of 0.149 cc/g, 15.274 Å, respectively. The nitrogen sorption curve (Figure 5) demonstrates hysteresis loop due to the mono and multilayer adsorption of nitrogen on the ONS4. The ramified hysteresis loop was observed from relative pressure (*p*/*p*_0_-value) of 0.5–0.9 which reflects condensation of nitrogen in different size inter-connected nanopores of NS. The pore size distribution curve exhibits that most of the pores were found to be in the range between 3.05 and 441.80 nm. In our results, ONS4 showed very low surface area, less pore size and volume probably due to use of ethylcellulose polymer in comparison to previously reported work on cyclodextrin nanosponges [55].

### 3.9. Cell Viability MTT Test

**Figure 6:**
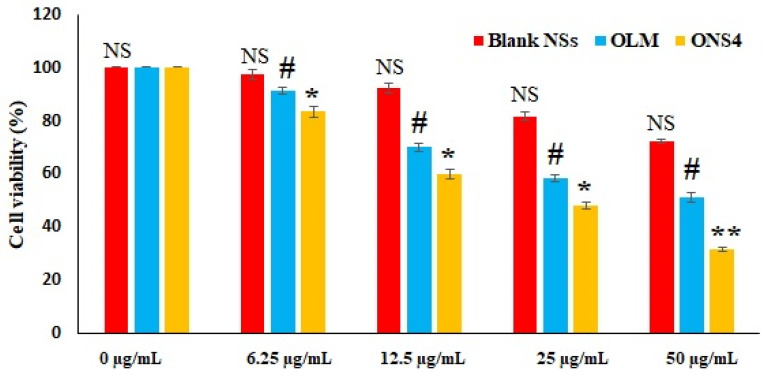
Comparative cell viability of blank NSs, pure OLM and OLM-loaded nanosponges (ONS4). * *p* < 0.005 highly significant—Compared between ONS4 vs. blank NSs and pure OLM at concentration of 50 µg/mL. ** *p* < 0.01 highly significant—Compared between the pure OLM and ONS4 carrier. # *p* < 0.05 significant—Compared between pure OLM vs. blank NSs at all concentrations. NS—Non-significant at all concentrations.

The chemical-colorimetric assay based on the MTT test exhibited concentration dependent reduction in the cell viability. This test measures the cellular metabolic activity, correlated with the darkness of the solution indicates greater metabolic activity and cell viability. The concentration versus percent cell viability data for the cells incubated with blank NSs, free OLM drug and the optimized NS carrier are presented in Figure 6. The optimized ONS4 carrier produced the lowest cell viability (31.44 ± 0.40%, 48.10 ± 1.21%, 59.91 ± 1.13% and 83.27 ± 1.10%) at concentrations of 50, 25, 12.5 and 6.25 µg/mL, respectively in comparison to free OLM (51.25 ± 1.12%, 58.42 ± 1.25%, 69.92 ± 1.36% and 90.39 ± 0.87%) and (72.29 ± 1.43%, 81.71 ± 1.67%, 92.07 ± 0.87% and 97.43 ± 1.76%) for the blank NSs prepared without any drug. The efficacy between ONS4 and the free OLM was much more marked at higher concentrations. The IC50 values for ONS4, OLM, blank-NSs were found to be 14.80 ± 1.21, 35.96 ± 0.45, 40.29 ± 1.17 µg/mL, respectively. IC50 is a half maximal inhibitory concentration, which represents 50% inhibition of the cell growth in vitro indicating the effectiveness of the cytotoxic entity. The optimized ONS4 carrier showed the significant cytotoxicity due to the sustained release of OLM and nano-range of NSs. It has been reported that RAS (renin-angiotensin system) and NF-κB (nuclear factor kappa-light-chain-enhancer of activated B cells) signaling is key factor for the survival of cancer cells, which causes therapeutic intervention. OLM blocks the RAS and NF-κB pathway [56] that could be the reason for significant cytotoxicity activity against A549 cell lines in our studies.

### 3.10. In Vivo Antihypertensive Studies

**Figure 7:**
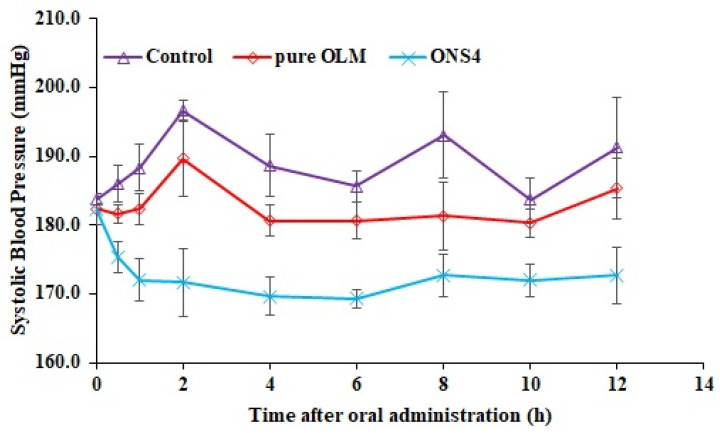
Systolic blood pressure lowering effect of the control, pure OLM and ONS4 samples after oral administration of in SHRs.

The systolic blood pressure (SBP) after oral administration of control, pure OLM and ONS4 was shown in Figure 7. The SBP was decreased significantly (*p* < 0.05) at each time point as compared to the control. The oral administration of the optimized nanosponge carrier (ONS4) reduced SBP highly significantly (*p* < 0.01) as compared to the control and the pure OLM drug. A sustained antihypertensive effect was observed in nanosponge carrier (ONS4) administered group during 12 h of the experiment, probably due to drug release retardation by ethylcellulose polymer. The maximum SBP lowering of pure OLM and ONS4 were observed as 180.3 ± 2.07 mmHg and 169.3 ± 1.37 mmHg at 10 h and 6 h, respectively. The OLM-loaded NSs (ONS4) controlled the SBP in hypertensive rats for prolong period of time by minimizing the limitation of oral delivery of OLM. The sustained release and reduced first pass metabolism by ethylcellulose polymer could be the reason of reduction in SBP.

These data suggest that when this formulation is administered orally, it can sustain the release of OLM in human body and will produce effective therapeutic action in hypertension and lung cancer, and simultaneously will reduce the side effects of OLM drug.

## 4. Conclusions

Ehtylcellulose-based OLM-loaded nanosponges (ONS1-ONS4) have been successfully developed using emulsion-solvent evaporation method and was further evaluated by DSC, FTIR, XRD, SEM and in vitro release studies. Based on preliminary characterization, OLM-loaded nanosponges (ONS4) was considered as the optimized formulation and was evaluated for morphology, porosity, in vitro MTT assay against A549 cell line and in vivo antihypertensive activity in rat model. The optimized nanosponges (ONS4) presented a better sustained release with improved antihypertensive efficacy and antitumor activity against A549 cell lines. Hence, it was concluded that the developed nanosponges benefits from its nanosize, porous nature and promises better therapeutic efficacy. The proposed study proved that OLM-loaded nanosponges could have promising application for the treatment of systolic blood pressure and lung cancer; however, further discoveries need to be done in this regard which deserves to be deeply explored.

## Figures and Tables

**Table 1:** Composition of OLM-loaded nanosponges.

Nanosponges	OLM (mg)	EC (mg)	PVA (*w*/*v*%)
ONS1	40	50	0.2
ONS2	40	100	0.2
ONS3	40	150	0.2
ONS4	40	200	0.2

**Table 2:** Characterization of developed OLM-loaded nanosponges.

Nanosponges	Physicochemical Properties *				
	PS ± SD (nm)	PDI	ζP ± SD (mV)	%EE ± SD	%DL ± SD
ONS1	381 ± 8.2 *	0.312 ± 0.02 *	−15.7 ± 1.6 *	87.1 ± 4.1	1.89 ± 0.15
ONS2	387 ± 9.5 *	0.446 ± 0.02 *	−19.0 ± 2.1 *	87.9 ± 5.2	1.61 ± 0.28
ONS3	404 ± 12.4 *	0.355 ± 0.05 *	−18.2 ± 1.2 *	88.6 ± 4.6	1.52 ± 0.16
ONS4	487 ± 12.8 *	0.386 ± 0.03 *	−18.1 ± 1.8 *	91.2 ± 1.9	0.88 ± 0.35

* Data are not statistically different (*p* > 0.05). Values are presented in mean ± SD (n = 3) in each column.

## Data Availability

The data presented in this study are available on request from the corresponding author.

## Supplementary Materials

The following are available online at https://www.mdpi.com/article/10.3390/polym13142272/s1. Figure S1: FTIR spectrum of the pure OLM and OLM-loaded nanosponge carriers. The main characteristic peaks of the pure OLM are shown with dotted vertical lines. These peaks are present, weakened or shifted in OLM-loaded nanosponges (ONS1-ONS4); Figure S2: Comparative XRD diffractograms of the pure OLM and OLM-loaded nanosponge; Figure S3: Profiles of different kinetic models for release mechanism of OLM from OLM-loaded nanosponges.

## References

[1] Sung G., Ferlay J., Siegel R.L., Laversanne M., Soerjomataram I., Jemal A., Bray F. (2021). Global cancer statistics 2020: GLOBOCAN estimates of incidence and mortality worldwide for 36 cancers in 185 countries. CA Cancer J. Clin..

[2] De Lima M.P.B., Ramos D., Freire A.P.C.F., Uzeloto J.S., de Silva B.L., Ramos E.M.C. (2017). Quality of life of smokers and its correlation with smoke load. Fisioter. Pesqui..

[3] Sabir F., Qindeel M., Zeeshan M., Ul Ain Q., Rahdar A., Barani M., González E., Aboudzadeh M.A. (2021). Onco-Receptors Targeting in Lung Cancer via Application of Surface-Modified and Hybrid Nanoparticles: A Cross-Disciplinary Review. Processes.

[4] Molina J.R., Yang P., Cassivi S.D., Schild S.E., Adjei A.A. (2008). Non-small cell lung cancer: Epidemiology, risk factors, treatment, and survivorship. Mayo Clin. Proc..

[5] Saab S., Zalzale H., Rahal Z., Khalifeh Y., Sinjab A., Kadara H. (2020). Insights into Lung Cancer Immune-Based Biology, Prevention, and Treatment. Front. Immunol..

[6] Jin C., Wang K., Oppong-Gyebi A., Hu J. (2020). Application of nanotechnology in cancer diagnosis and therapy - A mini-review. Int. J. Med. Sci..

[7] Prasad M., Lambe U.P., Brar B., Shah I., Ranjan M.J.K., Rao R., Kumar S., Mahant S., Khurana S.K., Iqbal H.M.N. (2018). Nanotherapeutics: An insight into healthcare and multi-dimensional applications in medical sector of the modern world. Biomed. Pharmacother..

[8] Ahmed M.M., Anwer M.K., Fatima F., Iqbal M., Ezzeldin E., Alalaiwe A., Aldawsari M.F. (2020). Development of ethylcellulose based nanosponges of apremilast: In vitro and in vivo pharmacokinetic evaluation. Latin Am. J. Pharm..

[9] Bano N., Ray S.K., Shukla T., Upmanyu N., Khare R., Pandey S.P., Jain P. (2019). Multifunctional nanosponges for the treatment of various diseases: A review. Asian J. Pharm. Pharmacol..

[10] Bayda S., Adeel M., Tuccinardi T., Cordani M., Rizzolio F. (2020). The history of nanoscience and nanotechnology: From chemical-physical applications to nanomedicine. Molecules.

[11] Sutradhar K.B., Amin M.L. (2014). Nanotechnology in Cancer Drug Delivery and Selective Targeting. ISRN Nanotechnol..

[12] Navya P.N., Kaphle A., Srinivas S.P., Bhargava S.K., Rotello V.M., Daima H.K. (2019). Current trends and challenges in cancer management and therapy using designer nanomaterials. Nano Converg..

[13] Ahmed M.M., Fatima F., Anwer M.K., Ansari M.J., Das S.S., Alshahrani S.M. (2020). Development and characterization of ethyl cellulose nanosponges for sustained release of brigatinib for the treatment of non-small cell lung cancer. J. Polym. Engn..

[14] Zhang Q., Honko A., Zhou J., Gong H., Downs S.N., Vasquez J.H., Fang R.H., Gao W., Griffiths A., Zhang L. (2020). Cellular Nanosponges Inhibit SARS-CoV-2 Infectivity. Nano Lett..

[15] Ahmed M.M., Fatima F., Anwer M.K., Ibnouf E.O., Kalam M.A., Alshamsan A., Aldawsari M.F., Alalaiwe A., Ansari M.J. (2021). Formulation and in vitro evaluation of topical nanosponge-based gel containing butenafine for the treatment of fungal skin infection. Saudi Pharm J..

[16] Krabicová I., Appleton S.L., Tannous M., Hoti G., Caldera F., Rubin Pedrazzo A., Cecone C., Cavalli R., Trotta F. (2020). History of Cyclodextrin Nanosponges. Polymers.

[17] Anwer M.K., Iqbal M., Ahmed M.M., Aldawsari M.F., Ansari M.N., Ezzeldin E., Khalil N.Y., Ali R. (2021). Improving the Solubilization and Bioavailability of Arbidol Hydrochloride by the Preparation of Binary and Ternary β-Cyclodextrin Complexes with Poloxamer 188. Pharmaceuticals.

[18] Théophile H., David X.R., Miremont-Salamé G., Haramburu F. (2014). Five cases of sprue-like enteropathy in patients treated by olmesartan. Dig. Liver Dis..

[19] Si S., Li H., Han X. (2020). Sustained release olmesartan medoxomil loaded PLGA nanoparticles with improved oral bioavailability to treat hypertension. J. Drug. Deliv. Sci. Technol..

[20] Anwer M.K., Jamil S., Ansari M.J., Iqbal M., Imam D., Shakeel F. (2016). Development and evaluation of olmesartan medoxomil loaded PLGA nanoparticles. Mat. Res. Innov..

[21] Gorain B., Choudhury H., Kundu A., Sarkar L., Karmakar S., Jaisankar P., Pal T.K. (2014). Nanoemulsion strategy for olmesartan medoxomil improves oral absorption and extended antihypertensive activity in hypertensive rats. Colloids Surf. B.

[22] Kang M.J., Kim H.S., Jeon H.S., Park J.H., Lee B.S., Ahn B.K., Moon K.Y., Choi Y.W. (2012). In situ intestinal permeability and in vivo absorption characteristics of olmesartan medoxomil in self-microemulsifying drug delivery system. Drug Dev. Ind. Pharm..

[23] Thakkar H.P., Patel B.V., Thakkar S.P. (2011). Development and characterization of nanosuspensions of olmesartan medoxomil for bioavailability enhancement. J. Pharm. Bioall. Sci..

[24] Jain S., Patel K., Arora S., Reddy V.A., Dora C.P. (2017). Formulation, optimization, and in vitro-in vivo evaluation of olmesartan medoxomil nanocrystals. Drug Deliv. Transl. Res..

[25] Prajapati S.T., Bulchandani H.H., Patel D.M., Dumaniya S.K., Patel C.N. (2013). Formulation and evaluation of liquisolid compacts for olmesartan medoxomil. J. Drug Deliv. Sci. Technol..

[26] Gunda R.K., Manchineni P.R., Dhachinamoorthi D. (2018). Design, development, and in vitro evaluation of sustained release tablet formulations of olmesartan medoxomil. MOJ Drug Des. Dev. Ther..

[27] Abd-Alhaseeb M.M., Zaitone S.A., Abou-El-Ela S.H., Moustafa Y.M. (2014). Olmesartan potentiates the anti-angiogenic effect of sorafenib in mice bearing Ehrlich’s ascites carcinoma: Role of angiotensin (1-7). PLoS ONE.

[28] Vassiliou S., Nkenke E., Lefantzis N., Ioannidis A., Yapijakis C., Zoga M., Papakosta V., Derka S., Nikolaou C., Vairaktaris E. (2016). Effect of Olmesartan on the Level of Oral Cancer Risk Factor PAI1. Anticancer Res..

[29] Gayathri E., Punnagai K., Chellathai D.D. (2018). Evaluation of Anticancer Activity of Olmesartan and Ramipril on A549 Cell. Biomed. Pharmacol. J..

[30] Rote A.R., Bari P.D. (2010). Spectrophotometric estimation of olmesartan medoxomil and hydrochlorothiazide in tablet. Indian. J. Pharm. Sci..

[31] (2013). Approved Product of OLMETEC® (Olmesartan Medoxomil). https://www.tga.gov.au/sites/default/files/auspar-olmesartan-medoxomil-130226-pi.pdf.

[32] Brunner H. (2002). The new oral angiotensin II antagonist olmesartan medoxomil: A concise overview. J. Hum. Hypertens..

[33] Kreutz R. (2011). Olmesartan/amlodipine: A review of its use in the management of hypertension. Vasc. Health Risk Manag..

[34] Alexander M.R., Madhur M.S., Harrison D.G., Dreisbach A.W., Riaz K. (2019). What is the global prevalence of hypertension (high blood pressure)?. Medscape.

[35] Anwer M.K., Mohammad M., Ezzeldin E., Fatima F., Alalaiwe A., Iqbal M. (2019). Preparation of sustained release apremilast-loaded PLGA nanoparticles: In vitro characterization and in vivo pharmacokinetic study in rats. Int. J. Nanomed..

[36] Anwer M.K., Iqbal M., Aldawsari M.F., Alalaiwe A., Ahmed M.M., Muharram M.M., Ezzeldin E., Mahmoud M.A., Imam F., Ali R. (2021). Improved antimicrobial activity and oral bioavailability of delafloxacin by self-nanoemulsifying drug delivery system (SNEDDS). Drug. Deliv. Sci. Technol..

[37] Anwer M.K., Iqbal M., Muharram M.M., Mohammad M., Ezzeldin E., Aldawsari M.F., Alalaiwe A., Imam F. (2020). Development of Lipomer Nanoparticles for the Enhancement of Drug Release, Anti-microbial Activity and Bioavailability of Delafloxacin. Pharmaceutics.

[38] Sinha P., Datar A., Jeong C., Deng X., Chung Y.G., Lin L. (2019). Surface Area Determination of Porous Materials Using the Brunauer–Emmett–Teller (BET) Method: Limitations and Improvements. J. Phy. Chem. C.

[39] Alshetaili A.S. (2021). Gefitinib loaded PLGA and chitosan coated PLGA nanoparticles with magnified cytotoxicity against A549 lung cancer cell lines. Saudi J. Biol. Sci..

[40] Michalowski C.B., Arbo M.D., Altknecht L., Anciuti A.N., Abreu A., Alencar L., Pohlmann A.R., Garcia S.C., Guterres S.S. (2020). Oral Treatment of Spontaneously Hypertensive Rats with Captopril-Surface Functionalized Furosemide-Loaded Multi-Wall Lipid-Core Nanocapsules. Pharmaceutics.

[41] Acosta E. (2009). Bioavailability of nanoparticles in nutrient and nutraceutical delivery. Curr. Opin. Colloid Interf. Sci..

[42] Lerche D., Sobisch T. (2014). Evaluation of particle interactions by in situ visualization of separation behavior. Colloids Surf. A.

[43] Rahdar A., Sargazi S., Barani M., Shahraki S., Sabir F., Aboudzadeh M.A. (2021). Lignin-Stabilized Doxorubicin Microemulsions: Synthesis, Physical Characterization, and In Vitro Assessments. Polymers.

[44] Rahdar A., Taboada P., Hajinezhad M.R., Barani M., Beyzaei H. (2019). Effect of tocopherol on the properties of Pluronic F127 microemulsions: Physico-chemical characterization and in vivo toxicity. J. Mol. Liq..

[45] Aloorkar N.H., Kulkarni A.S., Ingale D.J., Patil R.A. (2012). Microsponges as Innovative Drug Delivery Systems. Int. J. Pharm. Sci. Nanotechnol..

[46] Sharma N., Madan P., Lin S. (2016). Effect of process and formulation variables on the preparation of parenteral paclitaxel-loaded biodegradable polymeric nanoparticles: A co-surfactant study. Asian. J. Pharm Sci..

[47] Pandav S., Naik J. (2014). Preparation and In Vitro Evaluation of Ethylcellulose and Polymethacrylate Resins Loaded Microparticles Containing Hydrophilic Drug. J. Pharm..

[48] Maji R., Ray S., Das B., Nayak A. (2012). Ethyl Cellulose Microparticles Containing Metformin HCl by Emulsification-Solvent Evaporation Technique: Effect of Formulation Variables. Int. Schol. Res. Not..

[49] Pandi P., Bulusu R., Kommineni N., Khan W., Singh M. (2020). Amorphous solid dispersions: An update for preparation, characterization, mechanism on bioavailability, stability, regulatory considerations and marketed products. Int. J. Pharm..

[50] Perrin J.H. (1978). Sustained and Controlled Release Drug Delivery Systems.

[51] Wasilewska K., Winnicka K. (2019). Ethylcellulose–A Pharmaceutical Excipient with Multidirectional Application in Drug Dosage Forms Development. Materials.

[52] Trofimiuk M., Wasilewska K., Winnicka K. (2019). How to modify drug release in paediatric dosage forms? Novel technologies and modern approaches with regard to children’s population. Int. J. Mol. Sci..

[53] Mathew S.T., Devi S.G., KV S. (2007). Formulation and evaluation of ketorolac tromethamine-loaded albumin microspheres for potential intramuscular administration. AAPS PharmSciTech..

[54] Sharma R., Walker R.B., Pathak K. (2011). Evaluation of the Kinetics and Mechanism of Drug Release from Econazole nitrate Nanosponge Loaded Carbapol Hydrogel. Indian J. Pharm. Edu. Res..

[55] Sadjadi S., Heravi M.M., Daraie M. (2017). Cyclodextrin nanosponges: A potential catalyst and catalyst support for synthesis of xanthenes. Res. Chem. Intermed..

[56] Bakhtiari E., Hosseini A., Boroushaki M.B., Mousavi S.H. (2015). Synergistic, cytotoxic and apoptotic activities of olmesartan with NF-κB inhibitor against HeLa human cell line. Toxicol. Mech. Methods.

