# Supplementary material for: Design of Olmesartan Medoxomil-Loaded Nanosponges for Hypertension and Lung Cancer Treatments
Source: Polymers (Basel). 2021 Jul 11;13(14):2272. doi: 10.3390/polym13142272 (PMC8309359; doi:10.3390/polym13142272)
Supplement: Supplementary file 1 [file polymers-13-02272-s001.zip › polymers-1279688-supplementary.pdf]

polymers

Article

Design of Olmesartan Medoxomil-loaded Nanosponges for Hypertension and Lung Cancer Treatments

Bjad K. Almutairy [1], Abdullah Alshetaili [1], Amer S. Alali [1], Mohammed Muqtader Ahmed [1], Md. Khalid Anwer [1,*] and M. Ali Aboudzadeh [2,*]

[1] Department of Pharmaceutics, College of Pharmacy, Prince Sattam Bin Abdulaziz University, Alkharj 11942, Saudi Arabia; b.almutairy@psau.edu.sa (B.K.A.); a.alshetaili@psau.edu.sa (A.A.); a.alali@psau.edu.sa (A.A.); mo.ahmed@psau.edu.sa (M.M.A.)

[2] CNRS, University Pau & Pays Adour, E2S UPPA, Institut des Sciences Analytiques et de Physico-Chimie pour l'Environnement et les Matériaux, 64000 Pau, France

* Correspondence: mkanwer2002@yahoo.co.in (M.K.A); m.aboudzadeh-barihi@univ-pau.fr (M.A.A.)

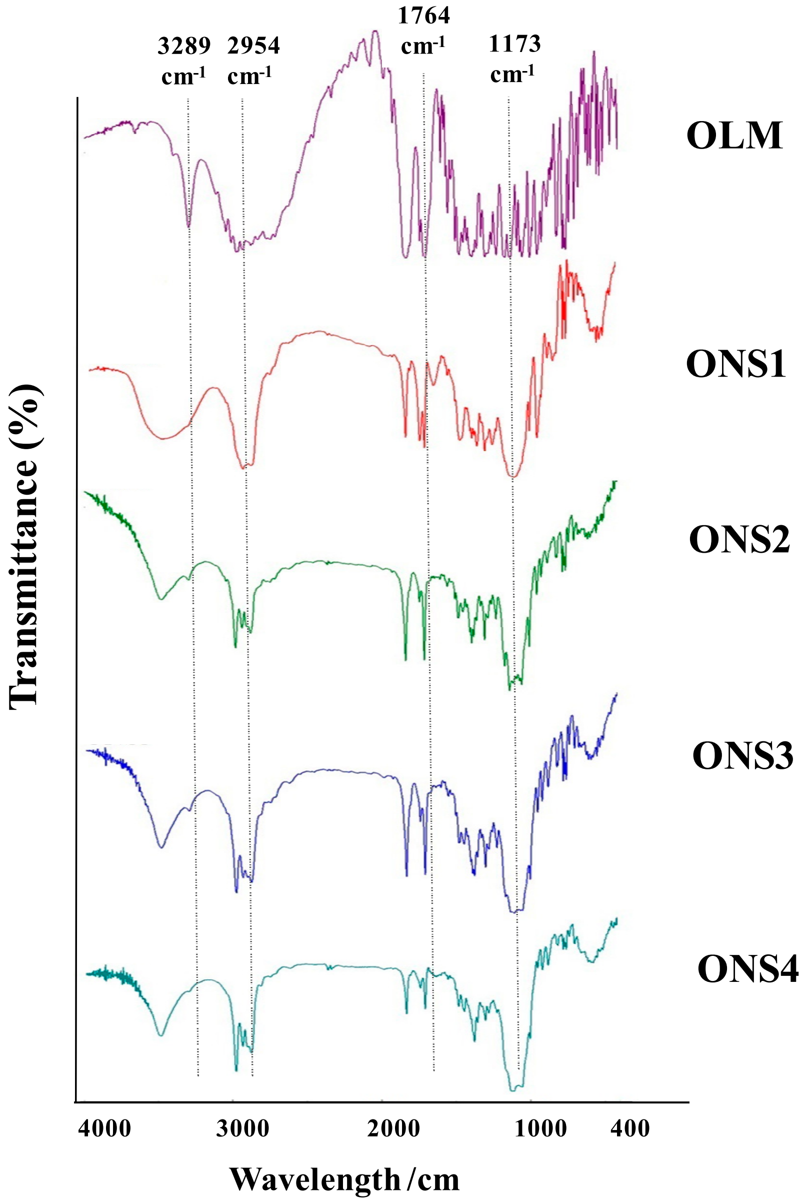

Figure S1. FTIR spectrum of the pure OLM and OLM-loaded nanosponge carriers. The main characteristic peaks of the pure OLM are shown with dotted vertical lines. These peaks are present, weakened or shifted in OLM-loaded nanosponges (ONS1-ONS4).

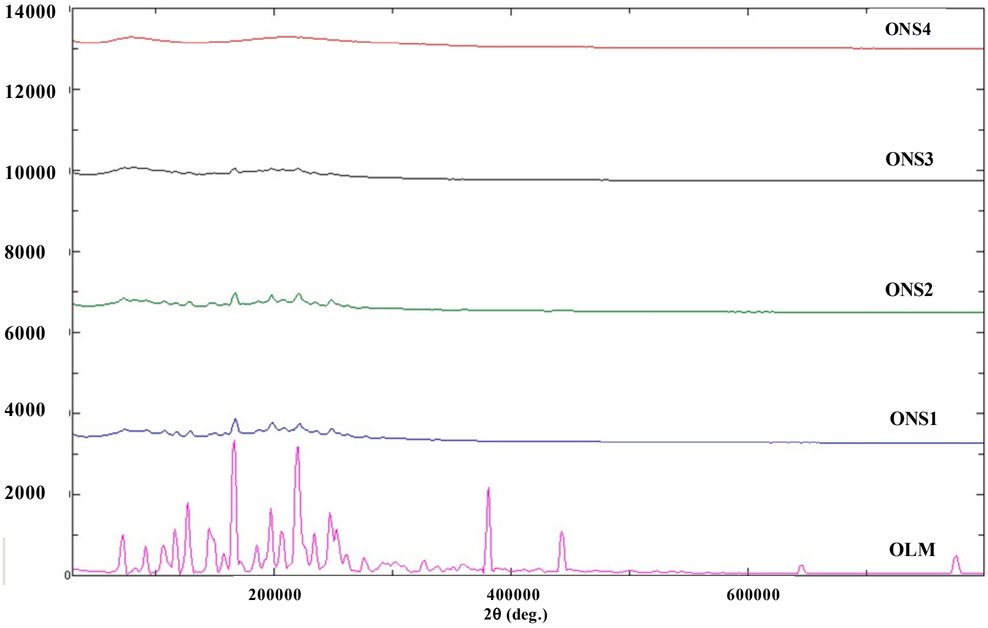

Figure S2. Comparative XRD diffractograms of the pure OLM and OLM-loaded nanosponge.

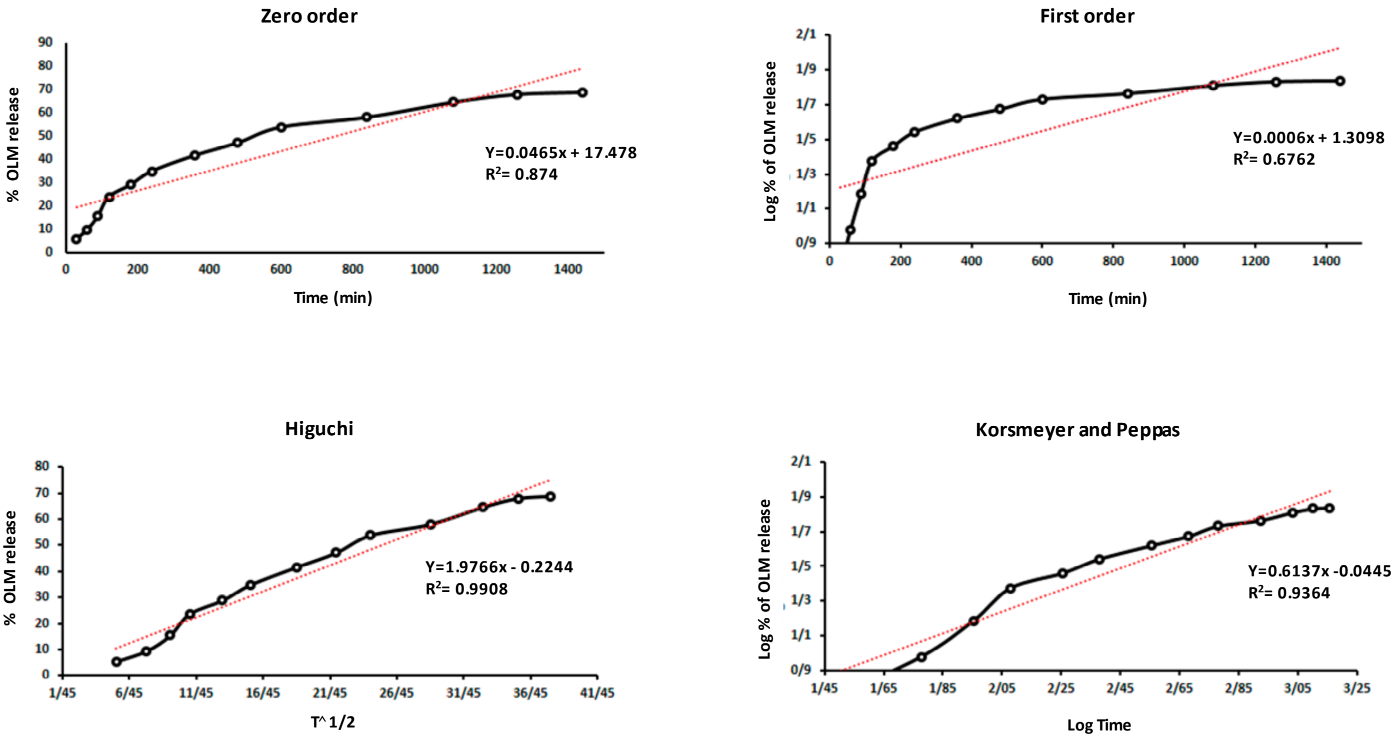

Figure S3. Profiles of different kinetic models for release mechanism of OLM from OLM-loaded nanosponges.
